# Laparoscopic reversal of Hartmann’s procedure: safety and feasibility

**DOI:** 10.1093/gastro/got018

**Published:** 2013-06-17

**Authors:** Daniel C.K. Ng, Salvatore Guarino, Steven L.C. Yau, Benny K.L. Fok, Hester Y.S. Cheung, Michael K.W. Li, C.N. Tang

**Affiliations:** ^1^Department of Surgery, Pamela Youde Nethersole Eastern Hospital, Hong Kong and ^2^Department of Surgery, Hong Kong Sanitorium Hospital

**Keywords:** Hartmann’s procedure, reversal, laparoscopy

## Abstract

**Aims:** The present study aimed to compare the surgical outcomes of patients receiving laparoscopic reversal of Hartmann’s procedure (RHP) with those receiving open surgery.

**Methods:** Records of all patients with RHP performed in our unit (including laparoscopic and open surgery) between 2000 and 2012 were retrieved. Data were retrospectively reviewed and compared.

**Results:** Eighty-two RHPs were performed between 2000 and 2012. Thirty-five were performed with an open approach and 47 with a laparoscopic approach. Conversion rate was 28% in the laparoscopic group. There was no difference, between the two groups, in operation time or blood loss. The median length of stay was significantly shorter in the laparoscopic group (12 vs 14 days, *P = *0.002) and fewer patients in the laparoscopic group had complications with post-operative paralytic ileus (2 vs 17%, *P = *0.038). None of the patients in the laparoscopic group developed incisional hernia at the conclusion of follow-up, as opposed to five in the open group (0 vs 14%, *P = *0.012).

**Conclusion:** Laparoscopic RHP is safe and feasible, with more favorable surgical outcomes, when compared with open surgery. Conversion rate is acceptable. It should be the technique of choice for patients undergoing RHP.

## INTRODUCTION

Reversal of Hartmann’s procedure (RHP) is a major undertaking that entails a long, midline, abdominal incision. Wound-related and pain-related complications are common and morbidity of 15–34% and peri-operative mortality up to 10% was reported [1].

Laparoscopic RHP has been increasingly practiced worldwide since the laparoscopic era. However, so far only a few studies have been published regarding the results of laparoscopic RHP. A recent review demonstrated its safety [2].

In this study, we reviewed the results of laparoscopic RHP performed in our unit. We evaluated all RHPs performed with either open or laparoscopic technique over the last 13 years and compared the peri-operative outcomes between the two.

## PATIENTS AND METHODS

This study aims to compare the surgical outcomes of patients receiving laparoscopic RHP with those receiving open surgery. Records of all patients with RHP performed in our unit between 2000 and 2012 were retrieved using electronic database and were retrospectively reviewed. Data including age, gender, indication of Hartmann’s procedure, interval to reversal, type of approach (open or laparoscopic), conversion rate (for laparoscopic group), operation time, blood loss, length of hospital stay and post-operative complications, were compared.

## SURGICAL TECHNIQUE

For patients undergoing open surgery, a midline incision was employed. For patients receiving laparoscopic RHP, a pure laparoscopic approach was used.

All patients underwent standard pre-operative assessment and preparation. They received oral bowel preparation the day before surgery and a per-rectal enema on the day of surgery. Antibiotics were administered by an anesthetist before skin incision.

The following is a description of the operative steps in laparoscopic RHP:

Patients are positioned in the Lloyd-Davis position. The chief surgeon and the camera assistant stand on the right side of the patient. A second assistant stands on the left side of the patient.

Pneumoperitoneum is achieved via a 12 mm trocar in the right flank, using an open technique. A second 5–12 mm trocar is inserted in the right iliac fossa and a variable number of 5 mm trocars are inserted in the right upper quadrant and suprapubic area as required.

After establishment of pneumoperitoneum, the peritoneal cavity is first assessed. Presence of dense adhesions or difficulty in identifying the rectal stump is an indication for early conversion. In the absence of these, the descending colon and splenic flexure are mobilized. The rectal stump is dissected out and identified.

Next, pneumoperitoneum is abolished and the end colostomy is mobilized and excised. The proximal colonic stump is delivered through the protected wound and the detachable anvil of a circular stapler is placed and anchored with a purse-string suture. The stump is then put back into the peritoneal cavity.

Finally, pneumoperitoneum is re-established and intra-corporeal colorectal anastomosis is performed using a circular stapler, inserted transanally. A covering ileostomy is constructed at the end of operation at the discretion of the operating surgeon.

## STATISTICAL ANALYSIS

Quantitative data were analysed using Mann-Whitney U-test. Qualitative data were analysed using chi-squared test or Fisher's exact test. Data were analysed using the intention-to-treat principle. The IBM SPSS Statistics Version 20 was used for analysis in all cases. A *P*-value of 0.05 or less was considered statistically significant.

## RESULTS

In a 13-year period, 82 RHPs were performed in our unit, comprising 50 male and 32 female patients. Thirty-five patients were operated on with an open approach and forty-seven with a laparoscopic approach. During 2000–2004, the majority (96%) of RHPs were performed with an open approach. From 2005 onwards, the majority (85%) of RHPs were performed using a laparoscopic approach. The median age was 60 (range: 32–90) in the open group and 61 (range: 34–84) in the laparoscopic group. No statistical difference was observed in the age and gender distribution between the two groups.

The initial procedure, i.e. Hartmann’s procedure, was performed as an emergency in 90% of cases. Table 1 shows the indications for Hartmann’s procedure in this series of patients. The median interval time from Hartmann’s procedure to reversal was 12 months (range: 5–45) in the open group and 14 months (range: 3–79) in the laparoscopic group (*P = *0.177).

**Table 1:** Indications for Hartmann’s procedure

	Open (*n* = 35)	Laparoscopic (*n* = 47)
Sigmoid volvulus	2	0
Perforated diverticulitis	9	13
Other perforations	0	3
Colonic malignancy	19	27
Gynecological malignancy	1	4
Anastomotic leakage	4	0

Thirteen patients (28%) in the laparoscopic group required conversion to open surgery. The reason for conversion was, in all cases, the presence of dense adhesions. Table 2 compares the operative data and outcomes in the two groups. There was no statistical difference in operation time or blood loss between the two groups. However, the post-operative hospital stay was significantly shorter in the laparoscopic group (*P = *0.002; Mann-Whitney U-test). Twenty patients required covering ileostomy in the open group, as opposed to only five patients in the laparoscopic group (*P = *0.040; chi-squared test).

**Table 2:** Comparison of operative data and outcomes

	Open	Laparoscopic	*P-*value
Median operation time^a^ (minutes)	153 (65–317)	167 (69–310)	0.854
Median blood loss^a^ (ml)	124 (30–800)	77 (10–550)	0.192
Median length of stay^a^ (days)	14 (6–35)	12 (6–19)	0.002
Covering ileostomy (%)	20 (57%)	5 (11%)	0.040

^a^median (range)

No 30-day mortality occurred in this series of patients. Table 3 compares the post-operative complications in the two groups. Two patients in the laparoscopic group required re-operation due to anastomotic complication. One patient in the open group required reoperation due to mesh infection. Compared with the open group, significantly fewer patients in the laparoscopic group (1 vs 6; *P = *0.038; chi-squared test) were complicated with post-operative paralytic ileus. At the conclusion of follow-up, five patients (14.3%) developed incisional hernia in the open group, whereas none of the patients in the laparoscopic group developed this complication (*P = *0.012; chi-squared test).

**Table 3:** Post-operative complications

	Open	Laparoscopic	*P-*value
Wound infection	8 (22%)	6 (13%)	0.251
Mesh infection^a^	1 (2.8%)	0 (0%)	0.573
Anastomotic complication^a^	0 (0%)	2 (4%)	0.505
Paralytic ileus	6 (17%)	1 (2%)	0.038
Pelvic collection	0 (0%)	1 (2%)	0.573
Incisional hernia	5 (14%)	0 (0%)	0.012
**Total**	**14 (40%)**	**10 (21%)**	**0.134**

^a^Required re-operation

## DISCUSSION

For almost a century, Hartmann’s procedure was the main surgical procedure for treatment of acute conditions affecting the left-side colon [3]. The restoration of gastrointestinal continuity after the initial surgery, i.e. RHP, has always represented a major challenge for surgeons and patients. The procedure is often difficult and involves painstaking dissection of peritoneal adhesions in the abdomen and pelvis via a major laparotomy wound. As a result, pain-related and wound-related morbidities are common. For this reason, some 40–50% of patients are considered unfit for RHP and are left with a permanent colostomy [4, 5].

The laparoscopic approach in colorectal surgery has been proven to result in a faster recovery and a shorter length of hospital stay, compared with the open approach [6]. There are a couple of reports on the use of the laparoscopic approach in RHP. A meta-analysis of comparative studies by Siddiqui *et al.* demonstrated the safety of the laparoscopic approach [2], with fewer complications and shorter hospital stay. Slawik and Dixon suggested that the laparoscopic approach had the added benefit of an easier splenic flexure mobilization [7]. Leroy *et al.* concluded that laparoscopic RHP was associated with a low conversion and complication rate when standardized operative protocol was followed and expert mentorship was available [8]. Van de Wall *et al.* reported that laparoscopic RHP had favorable outcomes [9], but a higher level of evidence was necessary to demonstrate its superiority to open surgery. Faure *et al.* demonstrated, in a comparative study, a shorter operating time, faster resolution of paralytic ileus and lower morbidity rate in laparoscopic RHP, when compared with open surgery [10].

The findings in this report were in keeping with those in the literature. While there was no difference in the operation time and intra-operative blood loss, significantly fewer patients in the laparoscopic group suffered from paralytic ileus, compared with the open group; this explains the significantly shorter hospital stay in the laparoscopic group. Additionally, the advantage of reduced wound-related complications, i.e. incisional hernia, following laparoscopic surgery was again demonstrated in this study.

It is interesting to note is that significantly fewer patients in the laparoscopic group required a covering ileostomy. The reason for this is unclear; however, laparoscopy may, by virtue of improved visualization through magnified view, help facilitate splenic flexure mobilization and allow a tension-free, well perfused anastomosis to be constructed more easily [7].

The wound infection rate was 22% in the laparoscopic group and 13% in the laparoscopic group. All wound infections occurred at the previous stoma site. From the results of a published paper concerning wound infection after colorectal surgery, the wound infection rate for closure of stoma is 34.6% [11]. The wound infection rate in this study compared favorably with the reported figure in the literature.

## CONCLUSION

This study confirms that laparoscopic RHP is safe and feasible, with more favorable surgical outcomes, compared with open surgery. Conversion rate is acceptable. In this era of minimal-access surgery and with increasing attention to fast-track protocols, we believe the laparoscopic approach should be the standard technique for patients undergoing reversal of Hartmann’s procedure.
